# Resveratrol targets PD-L1 glycosylation and dimerization to enhance antitumor T-cell immunity

**DOI:** 10.18632/aging.102646

**Published:** 2020-01-04

**Authors:** Sara Verdura, Elisabet Cuyàs, Eric Cortada, Joan Brunet, Eugeni Lopez-Bonet, Begoña Martin-Castillo, Joaquim Bosch-Barrera, José Antonio Encinar, Javier A. Menendez

**Affiliations:** 1 Program against Cancer Therapeutic Resistance (ProCURE), Metabolism and Cancer Group, Catalan Institute of Oncology, Girona, Spain; 2 Girona Biomedical Research Institute (IDIBGI), Girona, Spain; 3 Cardiovascular Genetics Centre, Department of Medical Sciences, University of Girona, Girona, Spain; 4 Centro de Investigación Biomédica en Red de Enfermedades Cardiovasculares (CIBERCV), Madrid, Spain; 5 Medical Oncology, Catalan Institute of Oncology, Girona, Spain; 6 Department of Medical Sciences, Medical School University of Girona, Girona, Spain; 7 Hereditary Cancer Programme, Catalan Institute of Oncology (ICO), Bellvitge Institute for Biomedical Research (IDIBELL), L'Hospitalet del Llobregat, Barcelona, Spain; 8 Hereditary Cancer Programme, Catalan Institute of Oncology (ICO), Girona Biomedical Research Institute (IDIBGI), Girona, Spain; 9 Department of Anatomical Pathology, Dr. Josep Trueta Hospital of Girona, Girona, Spain; 10 Unit of Clinical Research, Catalan Institute of Oncology, Girona, Spain; 11 Institute of Research, Development and Innovation in Biotechnology of Elche (IDiBE) and Molecular and Cell Biology Institute (IBMC), Miguel Hernández University (UMH), Elche, Spain

**Keywords:** PD-L1, resveratrol, immunotherapy, T cells, glycosylation

## Abstract

New strategies to block the immune evasion activity of programmed death ligand-1 (PD-L1) are urgently needed. When exploring the PD-L1-targeted effects of mechanistically diverse metabolism-targeting drugs, exposure to the dietary polyphenol resveratrol (RSV) revealed its differential capacity to generate a distinct PD-L1 electrophoretic migration pattern. Using biochemical assays, computer-aided docking/molecular dynamics simulations, and fluorescence microscopy, we found that RSV can operate as a direct inhibitor of glyco-PD-L1-processing enzymes (α-glucosidase/α-mannosidase) that modulate *N*-linked glycan decoration of PD-L1, thereby promoting the endoplasmic reticulum retention of a mannose-rich, abnormally glycosylated form of PD-L1. RSV was also predicted to interact with the inner surface of PD-L1 involved in the interaction with PD-1, almost perfectly occupying the target space of the small compound BMS-202 that binds to and induces dimerization of PD-L1. The ability of RSV to directly target PD-L1 interferes with its stability and trafficking, ultimately impeding its targeting to the cancer cell plasma membrane. Impedance-based real-time cell analysis (xCELLigence) showed that cytotoxic T-lymphocyte activity was notably exacerbated when cancer cells were previously exposed to RSV. This unforeseen immunomodulating mechanism of RSV might illuminate new approaches to restore T-cell function by targeting the PD-1/PD-L1 immunologic checkpoint with natural polyphenols.

## INTRODUCTION

Unlike immunologically “hot” tumors such as lung cancer, melanoma, and bladder cancer, most breast carcinomas are not inherently immunogenic. Consequently, they typically exhibit low T-cell infiltration and are unlikely to benefit from immune checkpoint-centric therapies [1–5]. Exceptions to this immunologically “cold” rule of breast carcinomas are the so-called basal-like [6–14] and HER2-positive [15–20] subtypes, both of which show evidence of immunogenicity including tumor immune infiltrates and stromal and intratumoral tumor-infiltrating lymphocytes, a good predictive marker for responses to immunotherapy. Correspondingly, the otherwise rare expression in most breast carcinomas of programmed death ligand-1 (PD-L1) – an archetypal immunosuppressive molecule on cancer cells that engages its receptor PD-1 on T-cells to suppress T-cell-mediated immune surveillance [21–23] – is markedly enriched in basal-like and HER2-positive tumors, thereby implying that PD-L1 confers a survival advantage in the tumor microenvironment (TME) of these specific breast cancer subtypes. However, the response rates reported in clinical trials for breast cancer with PD-1/PD-L1 checkpoint inhibitors as single agents have been rather disappointing (5–10%), although better clinical activities (up to 30%) and durable overall responses have been observed in patients with basal-like/HER2+ breast cancer and positive expression of PD-L1 [24–29]. Accordingly, the identification of new strategies to block the immune-inhibitory signal of PD-L1 in basal-like and HER2-positive subtypes is urgently needed.

In recent years, the cancer immunometabolism field has started to provide important insights into the pivotal role of metabolism in controlling immune cell function [30–35]. Indeed, much is now known about how the phenotypic switching of T-cells to be effective against tumor cells necessarily requires metabolic specialization, and how specific metabolic activities and tumor-driven shifts in the abundance of specific metabolites shape local immunosuppression and reduce the metabolic fitness of tumor-infiltrating lymphocytes. Indeed, the inhibitory nature of the metabolic interplay between tumor and immune cells in the TME supports its suitability as a target to overcome the immune escape of cancer cells and circumvent immunotherapy resistance. Accordingly, various combinations of immunotherapies with metabolic agents aimed to rewire T-cell fitness – by suppressing the immunosuppressive metabolic traits within the TME – are being tested in clinical trials [36–39]. Nonetheless, the appraisal of cancer cell-autonomous metabolic reprogramming as a *bona fide* driver of immune checkpoint signaling in tumor cells is a largely neglected area in cancer immunometabolism. Indeed, we are lacking even a minimal understanding of how pharmacological manipulations targeting core metabolic checkpoints such as AMPK, mTOR, and SIRT1, might fine-tune the expression of immune checkpoint receptors in cancer cells.

Here, we took advantage of the JIMT-1 cell line, a unique model of highly-aggressive basal-like/HER2-positive breast cancer naturally overexpressing the immunosuppressive molecule PD-L1 with 100% of the cells positive for PD-L1 [40], to explore the potential regulatory effects of mechanistically diverse metabolism-targeting drugs on PD-L1. By combining biochemical, computational, and microscopy approaches with label-free monitoring of T-cell activation, we provide the first evidence that the dietary polyphenol resveratrol (RSV) can directly target PD-L1 glycosylation and dimerization to enhance anti-tumor T-cell immunity.

## RESULTS

### Resveratrol increases the electrophoretic mobility of PD-L1 protein

**Figure 1:**
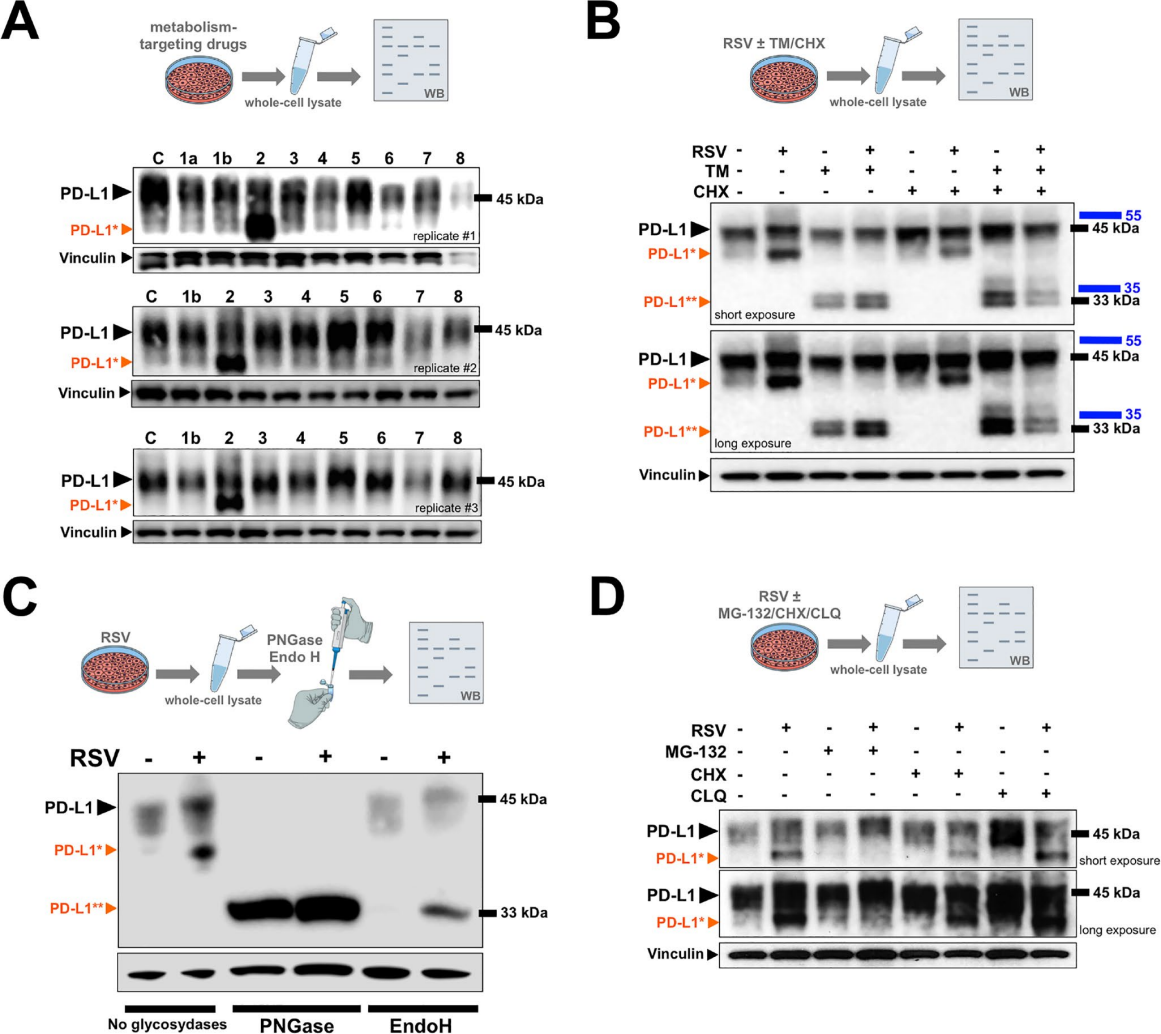
**PD-L1 is abnormally glycosylated in response to resveratrol.** (**A**) Representative immunoblot of PD-L1 protein in JIMT-1 cells cultured with or without metformin (1a), phenformin (1b), RSV (2), soraphen A (3), C75 (4), PP242 (5), Torin (6), AICAR (7), or compound C (8). (**B**, **D**) Representative immunoblots of PD-L1 protein in JIMT-1 cells cultured in the absence of presence of RSV, tunicamycin (TM), and/or cycloheximide (CHX), chloroquine (CLQ), and/or MG-132. (**C**) PD-L1 glycosylation patterns obtained from cell lysates of RSV-treated or untreated control cells that were further treated with PNGase F and Endo H and analyzed by western blotting (C: untreated control; black arrowhead, glycosylated PD-L1; *orange arrowhead, abnormal glycosylated PD-L1 form; **orange arrowhead, non-glycosylated PD-L1).

PD-L1 is a type I transmembrane glycoprotein with an apparent molecular weight of ~45 kDa. We first examined the protein expression of PD-L1 in JIMT-1 cells cultured with the following metabolism-targeting drugs: the SIRT1 agonist RSV, the mitochondrial complex I inhibitor phenformin, the acetyl-coA carboxylase inhibitor soraphen A, the fatty acid synthase inhibitor C75, and the AMPK/mTOR regulators AICAR, compound C, PP242, and Torin (Figure 1A). Based on three independent experiments aimed to reflect the substantial variation of PD-L1 expression by multiple parameters including cell density, both phenformin and AICAR consistently downregulated the major PD-L1 form at ~45 kDa. By contrast, treatment with RSV increased the electrophoretic mobility of PD-L1, which led to the conspicuous appearance of an intense immunoreactive band with a slightly lower molecular size (Figure 1A).

### Resveratrol disrupts N-linked glycosylation of PD-L1

Previous studies have unambiguously demonstrated that the ~45 kDa form of PD-L1 corresponds to the fully *N*-glycosylated mature protein [41–43]. As glycosylation of proteins often generates a heterogeneous migration pattern on immunoblots, such as that observed for PD-L1 in response to RSV treatment, we hypothesized that RSV might promote the accumulation of an aberrantly-glycosylated form of PD-L1. To test this, we re-evaluated the expression of PD-L1 in cells cultured with the antibiotic tunicamycin, which blocks *N*-linked glycosylation by inhibiting the enzyme *N*-acetylglucosamine phosphotransferase, responsible for the initial steps in protein glycosylation (Figure 1B). A significant portion of the major ~45-kDa PD-L1 band was reduced to ~33 kDa upon tunicamycin treatment in control (RSV-untreated) cell cultures, which is known to represent the non-glycosylated form of PD-L1. The molecular weight shift to non-glycosylated PD-L1 was more evident in cells co-treated with RSV and tunicamycin, and the addition of tunicamycin also prevented the accumulation of the lower PD-L1 band that appeared in response to RSV as a single agent (Figure 1B). Also, in the presence of the protein synthesis inhibitor cycloheximide, the turnover of the RSV-induced smaller species of PD-L1 and of the non-glycosylated (~33 kDa) form of PD-L1 appeared to be faster than that of the fully-glycosylated (~45 kDa) form.

Whole cell lysates obtained from JIMT-1 cells cultured in the absence or presence of RSV were further incubated with either *N*-glycanase (PNGase F), which removes all types of *N*-linked (asparagine-linked) glycosylation (i.e., high mannose, hybrid, bi-, tri-, and tetra-antennary) or endoglycosidase H (Endo H), which removes only high mannose and some hydrid types of *N*-linked oligosaccharides. Similar to the results with tunicamycin, PNGase F-driven removal of all *N*-linked glycan chains without regard to type completely blocked PD-L1 glycosylation irrespective of the absence or presence of RSV, as indicated by the conversion of the original pattern of PD-L1 post-translational glyco-modification to a discrete band corresponding to the non-glycosylated (~33 kDa) form (Figure 1C). By contrast, the addition of recombinant Endo H, which is known to cleave the high-mannose *N*-linked oligosaccharides found in the endoplasmic reticulum (ER), specifically prevented the aberrant glycosylated form of PD-L1 induced by the presence of RSV (Figure 1C).

Our results so far strongly suggest that the fraction of PD-L1 with a slightly lower molecular weight occurring in response to RSV is a non-fully, abnormally glycosylated form of PD-L1. Because PD-L1 protein stability is greatly influenced by its glycosylation state [41], we next determined the impact of the proteasome inhibitor MG-132 and the lysosomal inhibitor chloroquine on the RSV-induced faster migrating form of PD-L1. The abnormally glycosylated RSV-induced form of PD-L1 completely disappeared after treatment with MG-132 (Figure 1D), whereas chloroquine treatment notably protected the conspicuous PD-L1 migration pattern induced by RSV (Figure 1D).

### Resveratrol directly targets N-linked glycan decoration of PD-L1

**Figure 2:**
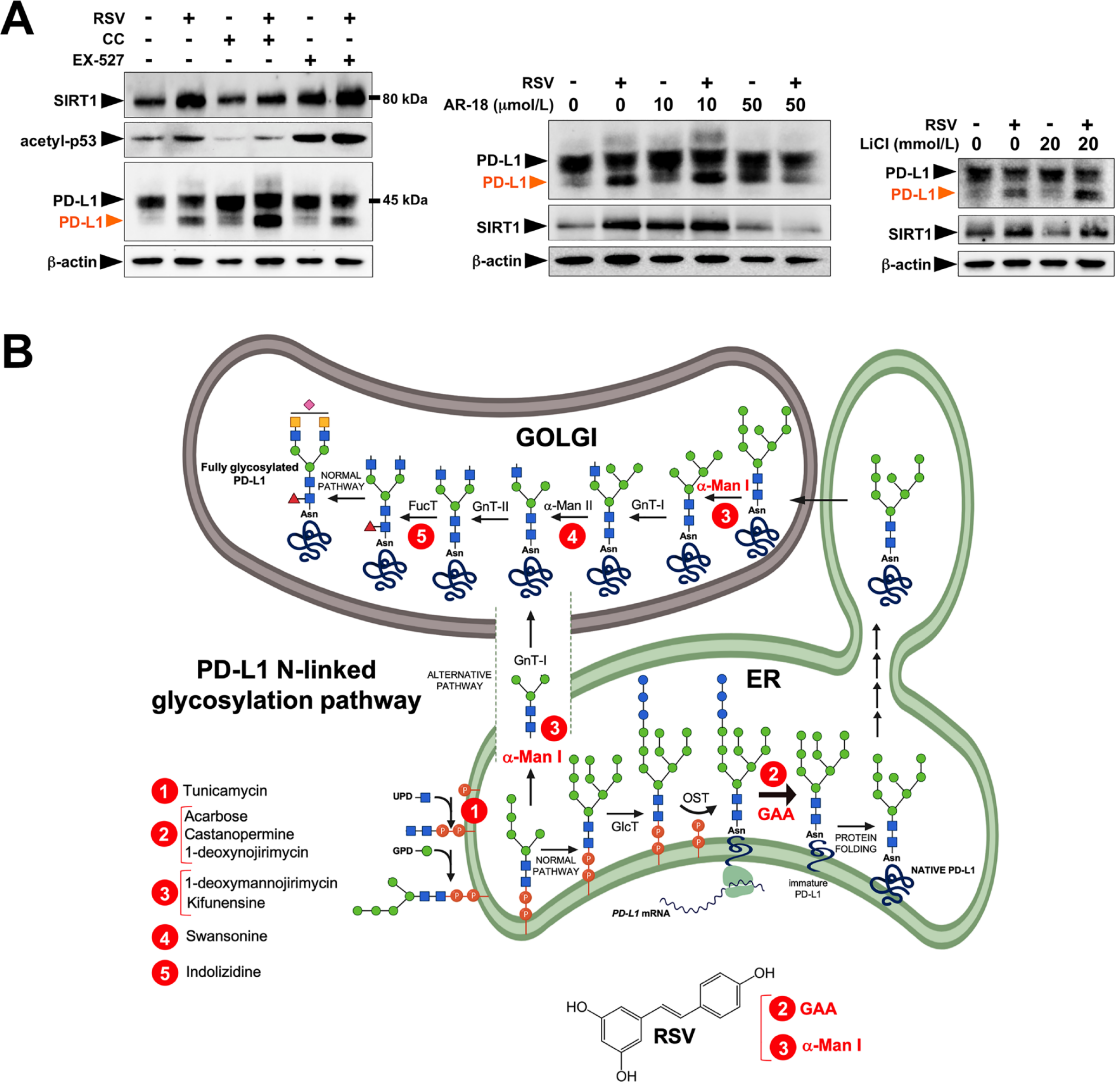
**Resveratrol alters PD-L1 *N*-glycosylation in a SIRT1-, AMPK-, and GSK3β-independent manner.** (**A**) Representative immunoblot of SIRT1, acetyl-p53 (Lys382), and PD-L1 in JIMT-1 cells cultured with or without RSV in the absence or presence of the AMPK inhibitor compound C, the SIRT1 inhibitor EX-527, and the GSK3β inhibitors AR-18 and LiCl. (**B**) Schematic representation of the biosynthesis and processing PD-1 *N*-linked glycosylation pathway, showing the sites of action of well-known glycoprotein-processing enzymes inhibitors. RSV is proposed to operate as a direct inhibitor of GAA and/or α-Man I enzymatic activities.

To substantiate that the RSV-induced aberrant glycosylated form of PD-L1 occurred concomitantly with the expected mode(s) of action of RSV, we first confirmed the well-known capacity of RSV to induce the endogenous expression of SIRT1 protein [42–44] (Figure 2A, *left panels*). Likewise, consistent with the ability of RSV to bind and inhibit histone HDAC enzymes [45, 46], RSV-treated JIMT-1 cells also showed increased p53 acetylation at lysine 382 [47]. The effects of RSV on PD-L1 glycosylation were neither prevented by the concurrent presence of the AMPK inhibitor compound C, nor by co-treatment with the SIRT1 inhibitor EX-527 [48] (Figure 2A, *left panels*). Moreover, the GSK3β inhibitors AR-18 (Figure 2A, *middle panels*) and LiCL (Figure 2A, *right panels*) failed to reverse the RSV-induced migration pattern of PD-L1, thereby ruling out the possibility that RSV might indirectly disrupt N-linked glycosylation through GSK3β activation [49] (Figure 2A, *right panels*).

**Figure 3:**
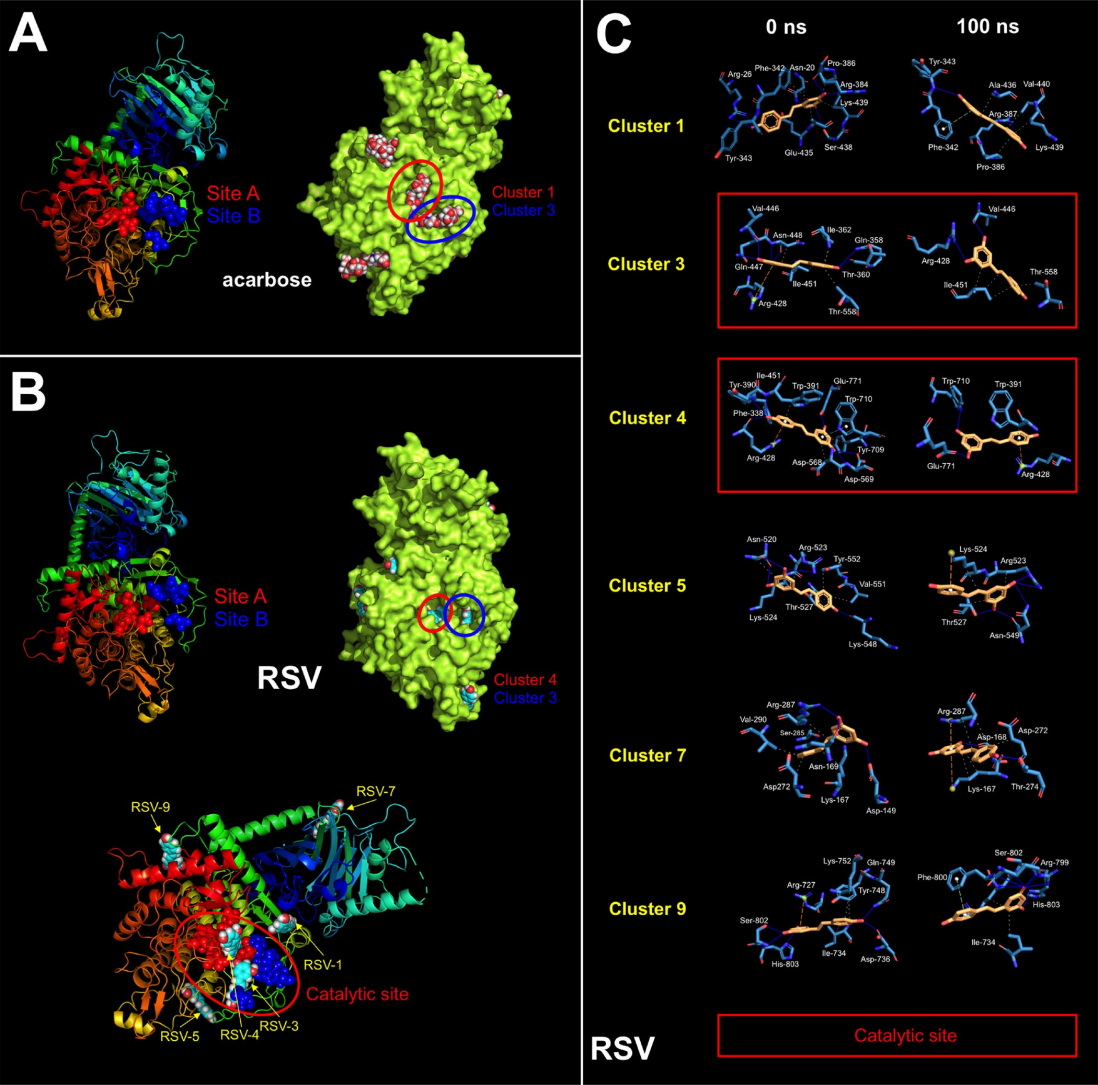
**Resveratrol is predicted to bind the catalytic site of yeast GAA.** Surface and backbone representations of yeast GAA showing the computationally predicted location of acarbose (**A**) and RSV (**B**) clusters. “Site A” refers to the pocket containing the proposed catalytic residues of yeast GAA whereas “site B” refers to a second cavity roughly 12 Å away from the active site pocket of yeast GAA [57]. (**C**) A detailed map of the molecular interactions of RSV in each cluster before (0 ns) and after 100 ns of molecular dynamics simulation. Each inset shows the detailed interactions of each RSV cluster docked to yeast GAA using the PLIP algorithm [124], indicating the participating amino acids involved in the interaction and the type of interaction (hydrogen bonds, hydrophilic interactions, salt bridges, Π-stacking, etc). Figures were prepared using PyMol 2.3 software.

**Figure 4:**
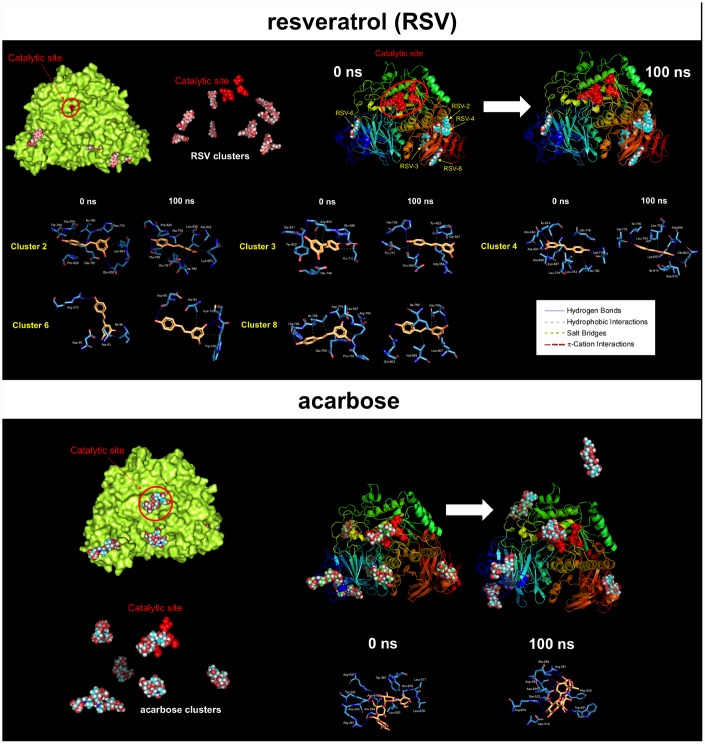
**Resveratrol is predicted to bind allosteric sites in the surface of human GAA.** Surface and backbone representations of human GAA showing the computationally-predicted location of RSV (*top panels)* and acarbose (bottom *panels*) clusters. A detailed map of the molecular interactions of RSV and acarbose in each cluster before (0 ns) and after 100 ns of molecular dynamics simulation. Each inset shows the detailed interactions of each RSV/acarbose cluster docked to human GAA using the PLIP algorithm [124], indicating the participating amino acids involved in the interaction and the type of interaction (hydrogen bonds, hydrophilic interactions, salt bridges, Π-stacking, etc). Figures were prepared using PyMol 2.3 software.

**Figure 5:**
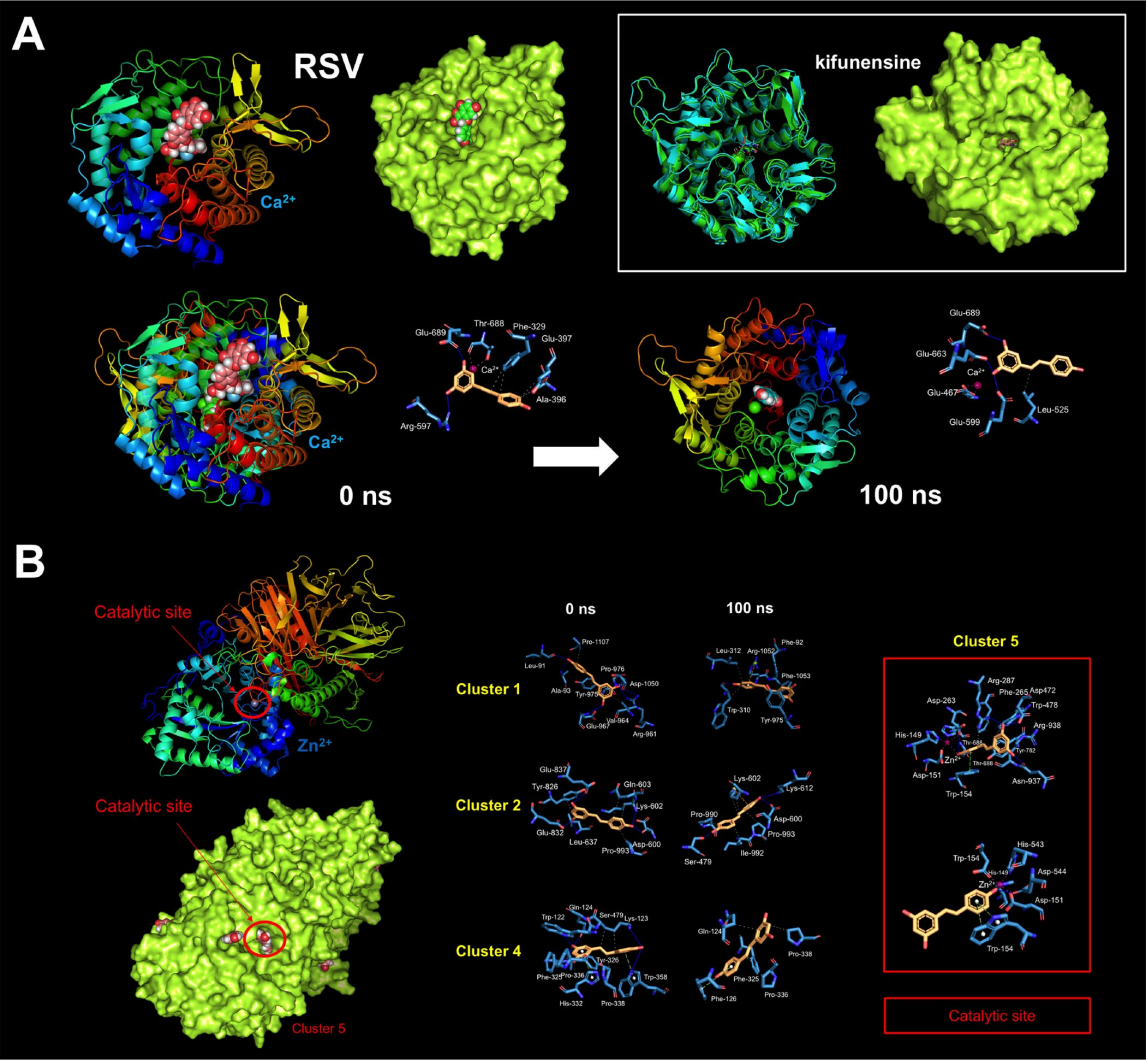
**Resveratrol is predicted to bind the catalytic site of human α-mannosidases.** Surface and backbone representations of human α-Man I (**A**) and homology model of human α-Man II (**B**) showing the computationally predicted location of RSV clusters. A detailed map of the molecular interactions of RSV in each cluster before (0 ns) and after 100 ns of molecular dynamics simulation. Each inset shows the detailed interactions of each RSV cluster docked to human GAA using the PLIP algorithm [124], indicating the participating amino acids involved in the interaction and the type of interaction (hydrogen bonds, hydrophilic interactions, salt bridges, Π-stacking, etc). The white inset in A shows a surface and backbone representations of human α-Man I docked to the α-Man I inhibitor kifunensine. Figures were prepared using PyMol 2.3 software.

These findings are consistent with a direct (and SIRT1-, AMPK-, GSK3β-independent) effect of RSV on the PD-L1 *N*-linked glycosylation machinery (Figure 2B). Indeed, RSV treatment notably mimicked the ability of inhibitors blocking *N*-linked (but not O-linked) glycosylation (e.g., swainsonine, castanospermine, kifunensin, or 1-deoxymannojirimycin) to alter the migration pattern of glycosylated PD-L1 on SDS-PAGE [50]. Specifically, the capacity of RSV to operate as an antiglycation agent *via* direct inhibition of α-glucosidase I (GAA) [51–54] and/or α-mannosidase I (α-Man I) [55, 56] would suffice to explain the ability of RSV to generate an Endo H-sensitive, high-mannose *N*-linked glycan form of PD-L1 in the ER (Figure 2B). In an attempt to provide a computational explanation for the inhibitory activity of RSV against GAA, we performed molecular docking and molecular dynamics (MD) assays of RSV against high-resolution crystal structures of the yeast (4J5T [57]) and human (5NN4 [58]) forms of GAA. RSV was predicted to occupy the catalytic site of yeast GAA with a higher binding energy than that predicted for acarbose, a well-known competitive inhibitor of GAA [59–63] (Figure 3A–3C; Supplementary Figure S1; Supplementary Table S1). Conversely, several RSV clusters were predicted to interact with enzymatic pockets distant to the active site of human GAA (Figure 4, top panels), which was accurately predicted to be occupied by acarbose (Figure 4, bottom panels; Supplementary Table S2). When we extended the *in silico* studies to α-Man I (i.e., ER 1,2-α-mannosidase (5KIJ) [64]), the sole RSV cluster predicted to interact with α-Man was found to occupy the catalytic site of the enzyme and exhibited a binding energy even higher than that predicted for kifunensin, a well-known pharmacological inhibitor of α-Man I [65, 66] (Figure 5A; Supplementary Table S3 ). Although the generation of a homology model of α-Man II predicted the ability of numerous clusters of RSV to interact with several enzymatic pockets including the active site (Figure 5B), the energy binding was lower than that predicted for α-Man I. Considering both the trajectories and the solvation/binding free energy differences (Supplementary Figure S2) of the different RSV-enzyme complexes following MD simulations up to 100 ns, the computational behavior of RSV was compatible with that of a non-competitive inhibitor bound to allosteric sites in the case of human GAA and of a competitive inhibitor capable of stably occupying the catalytic site in the case of human α-Man I.

### Resveratrol inhibits the cell membrane localization of glycosylated PD-L1

**Figure 6:**
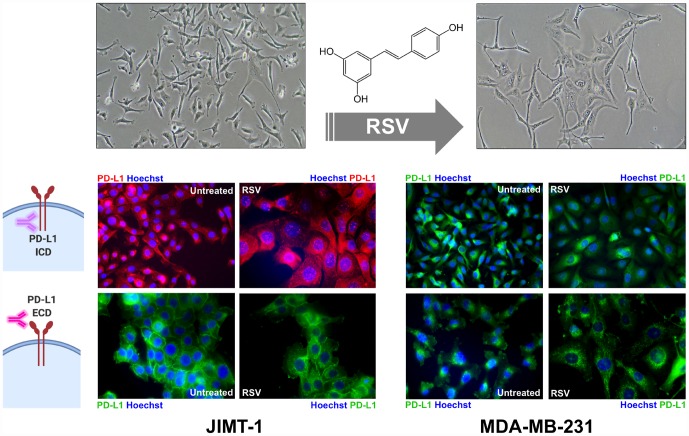
**Resveratrol alters the sub-cellular expression pattern of PD-L1.** Representative immunofluorescence staining of PD-L1 in JIMT-1 *(left panels*) and MDA-MB-231 (*right panels*) breast cancer cells cultured in the absence or presence of RSV, using an antibody directed against either an intracellular epitope (*top panels*) or an extracellular domain epitope (*bottom panels*) of PD-L1.

We next investigated whether the ability of RSV to target PD-L1 glycosylation altered PD-L1 membrane trafficking. Light microscopy observations suggested that RSV induced a hypertrophy-like phenotype in treated JIMT-1 and MDA-MB-231 cells (Figure 6, *top panels*), a second PD-L1-overexpressing breast cancer model representative of the basal-like subtype [40, 67, 68] as indicated by the transformation of spindle-shaped control cells to more enlarged, irregular, and flattened cell morphologies after switching to culture medium with RSV (Figure 6, *top panels*). Also, immunofluorescence microscopy using an antibody directed against an intracellular epitope of PD-L1 showed PD-L1 enrichment both in the plasma membrane and in specific cytoplasmic vesicular-like compartments (Figure 6, *bottom panels*). A more detailed analysis showed that cell membrane PD-L1 signals were clearly diminished in response to RSV, and this was accompanied by an increase in the number of mostly perinuclear aggresome-like, inclusion bodies. The RSV-driven cell membrane-to-cytoplasm redistribution of PD-L1 was more apparent when we used an antibody specifically directed to the extracellular domain of PD-L1, revealing an evident decrease in cell membrane-associated PD-L1 and the apparent retention of PD-L1 in perinuclear compartments with a punctate staining pattern (Figure 6, *bottom panels*).

### Resveratrol lowers the tumor cytotoxicity threshold to T-cells

**Figure 7:**
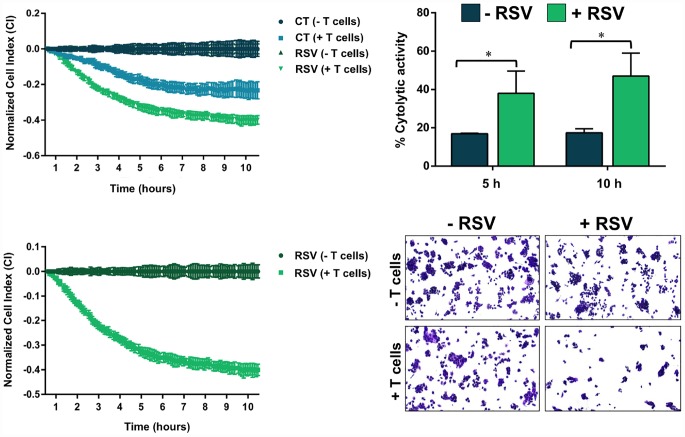
**Resveratrol enhances the susceptibility to T-cell-mediated tumor cell killing.** T-cell-mediated cell death of JIMT-1 cells pre-cultured in the absence of presence of RSV was measured using the xCELLigence system. Shown are the mean (±SD, n=3) of % lysis values calculated from the impedance-based lysis assay at 5 and 10 hours following the addition of T-cells. Also shown are microphotographs of representative T-cell-mediated cancer cell killing assays in which tumor cells were subjected to crystal violet staining. Statistical analysis was performed using GraphPad Prism 7, using two-way ANOVA with Sidak’s multiple comparison post-test, comparing untreated *versus* RSV-treated per time (* = *P* < 0.01).

Finally, we questioned whether RSV-induced abnormal glycosylation of PD-L1 would increase the susceptibility of basal-like/HER2+ breast cancer cells to T-cell elimination. To do this, we used an impedance- based assay (xCELLigence system) for real-time and label-free assessment of T-cell-mediated killing of PD-L1-expressing JIMT-1 cells [69, 71]. Briefly, impedance (or opposition) to an electric current occurs when adherent (tumor) cells bind to electrode plates; conversely, electrical impedance is reduced when tumor cells detach following killing, which can be measured by the real-time cell analyzer. The addition of T-cells in suspension over a monolayer of adherent tumor cells does not influence the impedance measurements as they do not contact with the electronic sensor; however, we could specifically detect T-cell activation events as the induction of T-cell-mediated tumor cell death results in morphological and detachment events that can readily be detected *via* changes in impedance. At a T-cell-to-cancer cell ratio of 5:1, we observed cytolytic activities as low as 15–17% following the addition of T-cells to JIMT-1 cells (Figure 7). When JIMT-1 cells were pre-treated with RSV before exposure to T-cells, we observed a significantly more robust cytolytic activity that reached >50% of the tumor cell population (Figure 7).

### RSV is predicted to target PD-L1 dimerization

**Figure 8:**
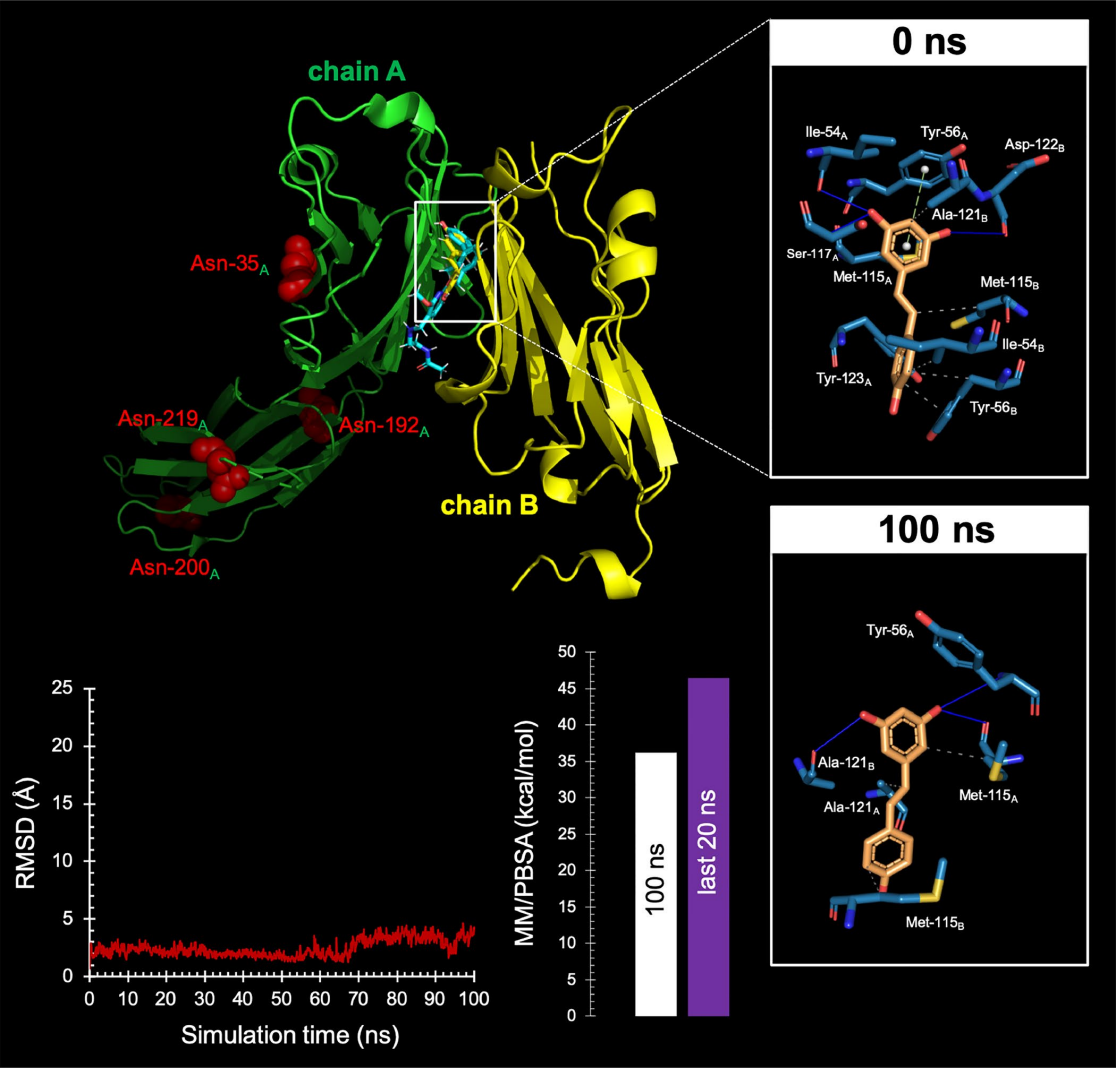
**Resveratrol is predicted to bind the PD-1 dimer interface.** *Top* Backbone representation of the PD-L1:PD-L1 dimer showing the computationally-predicted location of RSV (yellow) and BMS-202 (cyan). Chain A shows the location of the four Asn residues that can be glycosylated. The insets show the detailed maps of the molecular interactions of RSV with the amino acids at the hydrophobic pocket accommodating the BMS-202 inhibitor and formed at the PD-L1 dimer surface before (0 ns) and after 100 ns of molecular dynamics (MD) simulation, indicating the participating amino acids involved in the interaction and the type of interaction (hydrogen bonds, hydrophilic interactions, salt bridges, Π-stacking, etc). *Bottom*. *Left.* Trajectory of the RSV-forming complex with the PD-L1 dimer. *Right.* Molecular Mechanics/Poisson-Boltzmann Surface Area free energy analysis of the PD-L1 dimer forming a complex with RSV using YASARA dynamics v19.9.17 software. The best-docked complex as the initial conformation for MD simulation followed by 1000 snapshots (100 ns) obtained from the MD trajectory were employed to calculate the values of free energy binding of RSV. Additionally, the average value calculated for the last 200 snapshots (20 ns) is also displayed. YASARA-calculated binding energy provides positive values when the predicted binding is strong and stable whereas negative values indicate no binding. Figures were prepared using PyMol 2.3 software.

The strong exacerbation of the cytotoxic activity of T-cells against RSV-treated cancer cells raised the suspicion that additional PD-L1-targeted RSV mechanisms of action might be involved. We speculated that such non-mutually exclusive mechanisms might involve RSV binding to the dimerization surface of PD-L1, which is the same employed by PD-L1 to interact with PD-L1 [71–77]. Computer-aided docking/MD simulations predicted the capacity of RSV to occupy the cavity formed by two PD-L1 monomers (ΔG=-9.252 kcal/mol) in a manner closely mimicking that of BMS-202 (ΔG=-11.11 kcal/mol), a small-molecule capable of inhibiting the PD-1/PD-L1 interaction by inducing PD-L1 dimerization through the PD-1 interacting surface [71, 75] (Figure 8, *top*). The interacting mode of RSV with the dimeric PD-L1 complex included the key contribution of Ile54, Tyr56, Met115, Ala121, and Tyr123 residues on both monomers. An analysis of the RSV trajectory docked to the PD-L1 dimerization interface during a 100 ns period revealed a less than 5Å displacement compared with its initial position at the PD-L1 interaction interface (Figure 8, *bottom*). These findings, together with solvation binding energies as high as 45 kcal/mol, provided computational insight into the putative capacity of RSV to target PD-L1 dimerization and block the PD-1/PD-L1 interaction. When we examined monomeric and dimeric conformations of PD-L1 by native gel electrophoresis, the presence of appreciable amounts of PD-L1 dimer was apparent in RSV-treated cells whereas the monomeric form of PD-L1 was the major species in untreated control cells (Figure 8, *bottom*).

## DISCUSSION

**Figure 9:**
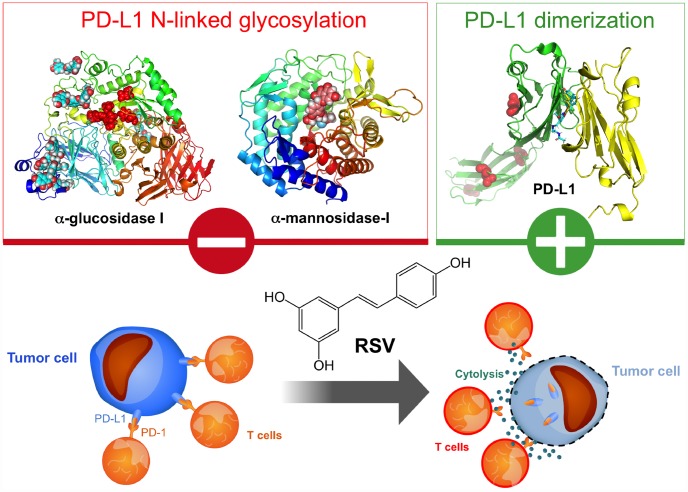
**Resveratrol enhances antitumor T cell immunity by promoting abnormal glycosylation and dimerization of PD-L1.** Post-translational modifications such as glycosylation, phosphorylation, palmitoylation or ubiquitination are essential for the folding, intracellular transport, and stabilization of the PD-L1 protein [41, 110–114]. PD-L1 is highly glycosylated, and *N*-linked glycosylation of PD-L1 critically maintains its protein stability and is required for its interaction with PD-1 to efficiently suppress T-cell activity. Recently, Bristol-Myers Squibb (BMS)-developed compounds with a common (2-methyl-4-biphenylyl)methanol scaffold have been reported to block the PD-1/PD-L1 interaction by interacting with the cavity formed by the two PD-L1 monomers and inducing the dimerization of PD-L1 [71–77]. Structural studies have revealed a dimeric protein complex with a single small molecule that stabilizes the dimer and thereby occludes the PD-1 interaction surface of PD-L1. We propose that RSV is a naturally occurring, double-strike PD-1/PD-L1 immune checkpoint inhibitor capable of directly blocking the enzymatic machinery in charge of the *N*-linked glycosylation of the nascent PD-L1 at the endoplasmic reticulum or directly binding to PD-L1 surfaces to induce PD-L1 dimerization and block PD-1 binding. This unforeseen ability of RSV to directly interfere with PD-L1 stability and trafficking impedes the correct targeting of PD-L1 to the cancer cell plasma membrane and ultimately elicits drastically enhanced cytotoxic T-lymphocyte immune-surveillance against tumor cells. These findings might illuminate new approaches to restore T-cell function by targeting the PD-1/PD-L1 immunologic checkpoint with natural polyphenols.

We provide the first demonstration that RSV targets the immune evasion capacities of cancer cells by directly disrupting *N*-glycan branching and promoting dimerization of PD-L1, thereby impeding the correct localization of PD-L1 to the plasma membrane, preventing the PD-1 interaction surface of PD-L1 and, consequently, increasing the susceptibility of biologically aggressive cancer cells to T-cell-mediated cell death (Figure 9).

The recently discovered ability of the mitochondrial I inhibitor metformin to target PD-L1 in cancer cells [67] provides support to the notion that the cancer cell-autonomous metabolic status can shape the composition of immune checkpoints in cancer cells. The activated form of AMPK generated in response to the metabolic crisis induced by metformin has been found to directly phosphorylate PD-L1 in a manner that promotes its abnormal glycosylation, resulting in ER accumulation and ER-associated protein degradation [39, 67]. We here characterized the unforeseen ability of RSV to disrupt *N*-linked glycosylation of PD-L1 and consequently reduce PD-L1 maturation and, ultimately, hamper its cancer cell surface-associated expression. Unlike metformin, however, the ability of RSV to generate a high mannose, abnormally glycosylated form of PD-L1 does not rely on its SIRT1-activating activity and also appears not to involve the well-known capacity of RSV to activate a positive feedback between AMPK and SIRT1 [78–80]. Rather, RSV likely operates as a direct inhibitor of glycoprotein-processing enzymes such as GAA and α-Man I (Figure 6), which trim terminal glucoses and mannose from nascent PD-L1, a crucial process for proper PD-L1 protein folding [81, 82]. The finding that the occurrence of the Endo H-sensitive abnormal glycosylated form of PD-L1 induced by RSV was diminished by the protein synthesis inhibitor cycloheximide, completely cleared by the proteasome inhibitor MG-132, and notably protected by the autophagy inhibitor chloroquine, altogether support a mechanism of action in which: 1) RSV might directly hinder GAA/α-Man I-driven trimming of glucoses and mannose immediately after the lipid-linked oligosaccharide precursor is transferred to Asn residues of nascent PD-L1 protein. Such a direct blockade of key glycoprotein-processing enzymes prevents the PD-L1 glycoprotein from attaining its native conformation because GAA and α-Man I ensure that, after correct folding, the processed (high-mannose) PD-L1 glycoprotein can move to the Golgi where *N*-linked glycans can be further remodeled; 2) The consequent imbalance between the amount of unfolded/misfolded PD-L1 protein in the ER lumen and the capacity of the ER machinery to refold the population of aberrantly glycosylated PD-L1 should result in ER stress *via* the integrated networks of aggresome, proteasome, and autophagy. Future studies are warranted to clarify whether, upon proteasome inhibition, the RSV-induced non-fully glycosylated (unstable) PD-L1 form might become polyubiquitinated [41] and undergo selective degradation *via* the autophagy-lysosomal pathway; and 3). Lastly, the glycosylation-targeted regulatory mechanism of RSV prevents the complete translocation of the fully-glycosylated population of PD-L1 to the cell membrane and, because the interaction between PD-L1 and PD-1 is modulated specifically by *N*-linked glycosylation [50], abrogates the binding of PD-1 to PD-L1, thereby providing a mechanism to switch on T-cell activity. Accordingly, impedance-based real-time analysis showed that cytotoxic T-lymphocyte activity was dramatically augmented when PD-L1-overexpressing JIMT-1 cancer cells were previously exposed to RSV.

Our findings might provide a foundation to exploit the *N*-glycan biosynthesis-targeted inhibitory activity of RSV as a new strategy to safely alter PD-L1 glycosylation *in vivo*. Although targeting the surface distribution of PD-L1 by modulating *N*-glycan branching is emerging as an exciting approach to boost the immune system against cancer cells [50, 83–85], the clinical development of currently existing inhibitors that impact processing or “trimming” of the glucosylated, high-mannose side chains by inhibiting one or more of the specific processing glycosidases/mannosidases, is still lacking regarding effective modifications capable of improving their therapeutic efficacy, selectivity, potency, and tolerability. Acknowledging the poor bioavailability (<1%) of orally-administered RSV [86, 87], the capacity of RSV-like strategies to target *N*-glycan branching of PD-L1 and, consequently, decrease the T-cell cytotoxicity threshold *via* targeting of glycoprotein-processing enzymes such as GAA and α-Man I might be a potential treatment option for cancer patients exhibiting a high neo-antigen, immunologically-active phenotype capable of attracting immune cells that ultimately triggers an unsuccessful tumor-immune cell interaction *via* PD-L1/PD-1 engagement [24–29, 88–90]. Early studies by Lucas et al. [91] showed that various colorectal and breast cancer cells (e.g., low PD-L1-expressing BT-474 and SK-Br3 cell lines) exposed to high concentrations of RSV upregulated PD-L1. The authors claimed that a potential upregulation of PD-L1 *in vivo* by RSV would render tumor cells more sensitive to immune checkpoints targeting PD-L1 [91], a counterintuitive hypothesis in terms of the potential chemopreventive value of RSV when considering that a putative upregulation of PD-L1 by RSV could enable pre-cancerous lesions to avoid antitumor immunity. Chin et al. [92] reported that the ability of the thyroid hormone L-thyroxin (T_4_) to inhibit the anti-cancer effects of RSV involves the up-regulation of PD-L1; conversely, RSV was found to down-regulate *PD-L1* expression. A complex interplay thus exists between RSV and PD-L1 that might present mutually antagonistic effects and might differentially occur in settings where baseline PD-L1 is low or high [92, 93]. Baseline PD-L1 expression differs according to the molecular phenotype of breast carcinomas, with the highest expression occurring in those with basal-like traits and the lowest expression in those with luminal traits [40, 43, 44]. Each breast cancer subentity responds differentially to extrinsic and intrinsic factors, and a significant heterogeneity in PD-L1 expression can be observed even within PD-L1-overexpressing breast cancer populations. Thus, whereas PD-L1 upregulation might represent a protective mechanism against RSV in low PD-L1-expressing cancer cells, constitutive PD-L1 ovexpressors could possess an inherent susceptibility to glycosylation defects under ER stress conditions imposed by GAA/α-Man-I inhibitors such as RSV. Accordingly, inhibition of *N*-linked glycosylation by RSV has been shown to trigger ER-mediated apoptosis in ovarian cancer cells [49]. Perhaps more importantly, RSV has been recently shown to induce immunogenic cell death and immune activation in terms of increased cytotoxic T-cells, and to potentiate the therapeutic outcomes of a PD-1 antibody in murine and human models of ovarian cancer [94]. These immunomodulatory actions of RSV in the tumor microenvironment, together with its ability to interfere with the glycosylation-driven stability of the immune checkpoint PD-L1, provides experimental evidence in support of combining RSV, which is virtually nontoxic with respect to its systemic effects, with immune checkpoint inhibitors, and might form the basis of future clinical trials.

Based on its mode of action involving core machineries (SIRT1 and AMPK) causally involved in the hallmarks of aging, such as epigenetic alterations, deregulated nutrient sensing, or mitochondrial dysfunction [62, 95], RSV has classically been considered an archetype member of the downstream type of calorie restriction mimetics (CRMs) including rapamycin, metformin, and polyamines (spermidine). Intriguingly, because normal human aging is characterized by a progressive decline in immune surveillance that is accompanied by PD-L1 upregulation to favor cancer initiation and progression even in the absence of a more complex mutational landscape [96–100], the suppression of PD-L1 signaling *via* direct targeting of PD-L1 glycosylation enzymes could represent a new immunometabolic mechanism through which RSV might prevent immune dysfunction and cancer development in the context of aging. Although there are conflicting data about whether the SIRT1 agonist activity of RSV might alleviate glucose intolerance in humans, it is reasonable to suggest that the ability of RSV to mimic acarbose in enhancing anti-cancer T-cell immunity [101, 102] uncovers an immunologic dimension to the previously observed capacity of RSV to exert anti-diabetic effects *via* direct inhibition of GAA [51–54, 103–105]. Acarbose, a pseudo-tetrasaccharide used to manage type 2 diabetes and a candidate drug in clinical trials targeting human aging based on its ability to improve health and lifespan in animal models [60–63, 106], is a potent competitive inhibitor of mammalian GAA, but is less effective against yeast GAA [51, 107]. RSV, however, has been shown to exert strong inhibition against both yeast and mammalian GAA activities [51, 53]. Although these discrepancies might be attributed to structural differences of the yeast and mammalian forms of the enzymes, we lack mechanistic insights capable of explaining the inhibition of GAA by RSV even at very low, clinically-relevant doses. In our hands, it was noteworthy that whereas RSV was *in silico* predicted to occupy the catalytic binding site of the yeast form of GAA with a higher binding energy than that predicted for acarbose, our computational model for human GAA predicted that the top binding modes of RSV localized distal to the catalytic residues in the active site pocket of GAA, thereby supporting a non-competitive mechanism *via* occupancy of GAA allosteric sites. Moreover, *in silico* modeling predicted the ability of RSV to operate as a kifunensine-like molecule capable of competitively occupying the catalytic site of the α-Man I enzyme, altogether offering an unforeseen mechanistic scenario linking the early recognized capacity of *N*-linked glycosylation to influence lifespan with the ability of RSV to directly block GAA and α-Man and activate an ER stress (dietary restriction-like) response [108, 55, 56]. Therefore, from an immunomodulatory perspective, our present findings propose that RSV might unexpectedly operate as a member of the upstream-type of CRMs, which employ a mechanism of action involving direct targeting of glucose metabolism (i.e., inhibition of class I glycosydases) and transmit a signal in the upstream direction to mimic CR.

Cancer immunomodulation involves the use of synthetic or natural agents capable of activating the immune response to impede tumor cell dissemination. The nutraceutical RSV, a natural polyphenolic phytoalexin that is present in red wine, red grape skin, berries, peanuts, and other natural sources, has recently been proposed as a cancer immunomodulatory molecule by either acting on immune cells or by sensitizing tumor cells to the cytotoxic effects of immune cells [109]. Our impedance-based real-time cell analysis showing that cytotoxic T-lymphocyte activity is dramatically exacerbated when highly aggressive cancer cells overexpressing PD-L1 in almost 100% of the cells were previously exposed to RSV strongly suggests that, beyond promoting abnormal PD-L1 glycosylation, RSV might be targeting the immunosuppressive signaling of PD-L1 by additional mechanisms. Computer-aided docking/MD simulations predicted the ability of RSV to locate at the center of the PD-L1 homodimer, filling a deep hydrophobic pocket that contributes multiple additional interactions between the PD-L1 monomers. Indeed, RSV was predicted to almost perfectly occupy the target space of Bristol-Myers Squibb (BMS)-developed nonpeptidic chemical inhibitors such as BMS-8, BMS-202, or BMS-1166, which have a common scaffold and interact with the cavity formed by two PD-L1 monomers, (consisting of Ile54, Tyr56, Met115, Ile116, Ala121, and Tyr123 [71, 77]), thereby blocking the PD-1/PD-L1 interaction by inducing PD-L1dimerization. The double-strike PD-1/PD-L1 immune checkpoint inhibitor-like behavior of RSV based on its ability to directly target PD-L1 either *via* key post-translational modifications such as *N*-linked glycosylation [110–114] or *via* direct binding to PD-L1 to block PD-1 binding [71–77] interferes with PD-L1 stability and trafficking, impedes the correct targeting of PD-L1 to the cancer cell plasma membrane, and lastly elicits considerably enhanced cytotoxic T-lymphocyte immune-surveillance against tumor cells (Figure 9). This unforeseen immunomodulating mechanism of RSV might provide new approaches to restore T-cell function by targeting the PD-1/PD-L1 immunologic checkpoint with natural polyphenols.

## MATERIALS AND METHODS

### Cell lines and culture conditions

JIMT-1 cells were obtained from the German Collection of Microorganisms and Cell Culture (Braunschweig, Germany) and grown in Dulbecco's modified Eagle's medium (DMEM) supplemented with 10% heat-inactivated fetal bovine serum (FBS; BioWhittaker Inc., Walkersville, MD), 1% L-glutamine, 1% sodium pyruvate, 50 U/mL penicillin, and 50 μg/mL streptomycin. MDA-MB-231 breast cancer cells were obtained from the American Type Culture Collection (Manassas, VA) and grown in Improved MEM (IMEM; BioSource International, Camarillo, CA) supplemented as above. All cells were maintained at 37°C in a humidified atmosphere of 95% air and 5% CO_2_. Cells were screened periodically for *Mycoplasma* contamination.

Approximately 250,000 cells were seeded in 60-mm dishes and treated with 100 μmol/L RSV (R5010), 5 mmol/L metformin (D150959), 100 μmol/L phenformin (P7045) (all from Sigma-Aldrich, St Louis, MO), 100 nmol/L soraphen A (kindly provided by Drs. Klaus Gerth and Rolf Jansen, Hemholtz Zentrum für Infektionsforschung GmbH, Braunschweig, Germany), 5 μg/mL C75 (C5490; Sigma-Aldrich), 200 nmol/L PP242 (S2218; Selleckchem, Houston, TX), 200 nmol/L Torin 2 (S2817; Selleckchem), 500 μmol/L AICAR (Cat. # sc-200659; Santa Cruz Biotechnology, Santa Cruz, CA), 5 μmol/L compound C (S7840, Selleckchem), 5 μg/mL tunicamycin (sc-3506, Santa Cruz Biotechnology), 20 μmol/L cycloheximide (sc-3508, Santa Cruz Biotechnology), 1 μmol/L MG-132 (S2619, Selleckchem), 10 μmol/L chloroquine (C6628, Sigma-Aldrich), 10 μmol/L EX-527 (S1541, Selleckchem), 10–50 μmol/L AR-A014418 (S7435, Selleckchem), or 20 mmol/L LiCl (L5509, Sigma-Aldrich) as single agents or in combination, as specified. In parallel, the untreated cultures were used as controls.

### Western blotting

Following treatment with the above-mentioned drugs, cells were lysed in 2% SDS, 1% glycerol, and 5 mmol/L Tris-HCl, pH 6.8. Samples were sonicated for 1 min (under ice water bath conditions) with 2 s sonication and 2 s intervals to fully lyse cells and reduce viscosity. Alternatively, cell samples were extracted with lysis buffer (150 mmol/L NaCl, 50 mmol/L Tris-HCl pH 7.4, 1 mmol/L EDTA, 1% Triton-X 100, 1 mmol/L phenylmethylsulfonyl fluoride, 1 mmol/L Na_3_VO_4_) and then incubated in the presence or absence of either PNGase F (P0704S, 1,000 Units/reaction) or Endo H (P0702S, 2,000 Units/reaction) (both from New England Biolabs, Ipswich, MA) for 2 h at 37°C using 30 μg of total protein per reaction. Protein content was determined by the Bradford protein assay (Bio-Rad, Hercules, CA). Sample buffer was added to extracts and boiled for 4 min at 100°C. Equal amounts of cellular protein were electrophoresed on 12% SDS-PAGE gels, transferred to nitrocellulose membranes, and incubated with antibodies against PD-L1 (E1L3N^®^ XP^®^ Rabbit mAb #13684), SIRT1 (C14H14 Rabbit mAb #2496), or acetyl-p53 (Lys382) (antibody #2525) (all from Cell Signaling Technology, Danvers, MA), followed by incubation with a horseradish peroxidase-conjugated secondary antibody, and chemiluminescence detection. Vinculin (sc-25336; Santa Cruz Biotechnology; 1:10,000 dilution) and β-actin (Cat. # 66009-1-Ig, Clone #: 2D4H5; Proteintech Group, Inc., Rosemont, IL) were employed as controls for protein loading.

### Immunofluorescence

Cells were seeded onto glass coverslips, treated with RSV for 24 h and then fixed with 4% paraformaldehyde in phosphate buffered saline (PBS). Following fixation at room temperature (RT) for 5 min, cells were permeabilized with 0.1% Triton X100/PBS. The coverslips were then placed in the antibody solution (E1L3N^®^ XP^®^ Rabbit mAb #13684 1:1,00 dilution or PD-L1 extracellular domain specific E1J2J rabbit mAb #15165 1:1,00 dilution, both from Cell Signaling Technology) and incubated for 60 min at RT. Cells were washed and stained with a secondary antibody. Cell nuclei were counterstained with Hoechst 33342. Images were captured using an Eclipse 50i fluorescence microscope equipped with NIS-Elements imaging software (Nikon, Tokyo, Japan).

### Molecular docking

Acidic human α-glucosidase (UniProt code: Q16706, PDB code: 5NN4), human ER α-mannosidase I (UniProt code: Q9UKMT, PDB code: 5KIJ), *Saccharomyces cerevisiae* α-glucosidase (UniProt code: P53008, PDB code: 4J5T), and PD-L1 (UniProt code: Q9NZQ7, PDB code: 5J89) crystallographic structures were obtained from the Research Collaboratory for Structural Bioinformatics (RCSB) Protein Data Bank (PDB). In the case of the human α-mannosidase II (UniProt code: Q16706), which lacks a high-resolution available PDB structure, a homology model was generated employing the 1QWU structure (a 1.2-Å resolution structure for the Golgi α-Man II from *Drosophila melanogaster*) as a template. The specific edition of protein structures was made using PyMol software (PyMOL Molecular Graphics System, v2.3.3 Schrödinger, LLC, at http://www.pymol.org/) without further optimization.

Molecular docking experiments were carried out using YASARA v19.9.17 software executing the AutoDock 4 algorithm with AMBER99 as a force field [115–118]. Briefly, a total of 999 flexible docking runs were set and clustered (7 Å) around the putative binding sites, that is, two complexed compounds belong to different clusters if the ligand Root-Mean-Square Deviation of their atomic positions is greater than a minimum of 7 Å around certain hot spot conformations. The YASARA pH command was set to 5.0, 7.2, 7.2, and 6.4 when running the molecular docking simulations of acidic human α-glucosidase, human ER α-mannosidase I, *S. cerevisiae* α-glucosidase, and human Golgi α-mannosidase II homology model, respectively. The YASARA software calculated the Gibbs free energy variation (ΔG, kcal/mol), with more positive energy values indicating stronger binding. To calculate this parameter, Autodock Vina uses a force field scoring function that considers the strength of electrostatic interactions, hydrogen bonding between all atoms of the two binding partners in the complex, intermolecular van der Waals forces, and also solvation and entropy contributions [119]. All the values are included in the corresponding tables with a negative sign. Only the ΔG value for the best RSV docked in each cluster is shown. Dissociation constants were recalculated from the average binding energy of all RSVs of each cluster. The number of RSV docked molecules in each cluster was identified as “members” (in percentages).

### Molecular dynamics simulations

YASARA dynamics v19.9.17 was used for all the MD simulations with AMBER14 as a force field. The simulation cell was allowed to include 20 Å surrounding the protein and filled with water at a density of 0.997 g/mL. Initial energy minimization was carried out under relaxed constraints using steepest descent minimization. Simulations were performed in water at constant pressure-constant temperature (25°C) conditions. To mimic physiological conditions, counter ions were added to neutralize the system; Na^+^ or Cl^-^ were added in replacement of water to give a total NaCl concentration of 0.9% and pH was maintained at 7.4. Hydrogen atoms were added to the protein structure at the appropriate ionizable groups according to the calculated pKa in relation to the simulation pH (i.e., a hydrogen atom will be added if the computed pKa is higher than the pH). The pKa was computed for each residue according to the Ewald method [120]. All simulation steps were run by a preinstalled macro (md_run.mcr) within the YASARA suite. Data were collected every 10 ps. The molecular mechanics/ Poisson-Boltzmann surface area (MM/PBSA) was implemented with the YASARA macro md_analyzebindenergy.mcr to calculate the binding free energy with solvation of the ligand, complex, and free protein, as previously described [121–123].

All of the figures were prepared using PyMol 2.0 software and all interactions were detected using the PLIP algorithm [124].

### Human T-cell culture

To acquire activated T-cells, human peripheral blood mononuclear cells (Cat. #70025) were cultured in ImmunoCult-XF™ T cell expansion medium (Cat. #10981) containing ImmunoCult-XF™ Human CD3/CD28/CD2 T-cell activator (Cat. #10971) (all from StemCell Technologies, Vancouver, BC, Canada), and 10 ng/mL IL-2 (Cat. #200-02; PeproTech, Rocky Hill, NJ) for one week, as per the manufacturers’ instructions.

### Cytolytic T-cell assay

T-cell-mediated lysis of tumor cells was monitored using an impedance-based approach. One hundred microliters of DMEM containing 10% FBS was added to each well of an E-Plate 16 (Roche Applied Sciences, Indianapolis, IN). Background impedance was measured using the xCELLigence RTCA instrument (Roche) at 37°C and 5% CO_2_. Tumor cells were harvested, counted, and resuspended at a density of 5 × 10^4^ cells/mL in DMEM with 10% FBS, and 100 μL of the tumor cell suspension was added to each well of the E-Plate 16. Impedance was measured every 5 minutes for approximately 24 h as described. Media from E-Plate 16 wells were removed and replaced with unsupplemented culture media in control wells or media containing 100 μg/mL RSV in experimental wells, and impedance was measured every 5 minutes for approximately 24 h. T-cells were counted and resuspended at a concentration of 5 × 10^5^ cells/mL in DMEM including an antii-CD3 antibody (Cat. #16-0037, 100 ng/mL; eBioscience, Thermo Fisher Scientific Inc., Philadelphia, PA). One hundred microliters of the T-cell suspension or media alone was added to respective wells and impedance was measured every 5 minutes for an additional 24 h. T-cell-mediated cell death of tumor cells was monitored in real-time and indicated as a decrease in the so-called cell index (CI) obtained using RTCA Software 1.2 (Acea Biosciences, San Diego, CA). Results were normalized to 1 to 2 h following T-cell addition. Cytolytic activity was calculated as the percentage of cytolysis 5 and 10 hours after the normalization time (=[CI_no effector_-CI_effector_]/CI_no effector_ × 100).

### Statistical analysis

Cytolytic activity means were compared using a two-way ANOVA with Sidak’s multiple comparison post-test. Results were designated significant when the *P*-value was < 0.01.

## Supplementary Material

Supplementary Figures

Supplementary Tables
