# Supplementary material for: Resveratrol targets PD-L1 glycosylation and dimerization to enhance antitumor T-cell immunity
Source: Aging (Albany NY). 2020 Jan 4;12(1):8–34. doi: 10.18632/aging.102646 (PMC6977679; doi:10.18632/aging.102646)
Supplement: Supplementary Figures [file aging-12-102646-s002..pdf]

SUPPLEMENTARY FIGURES

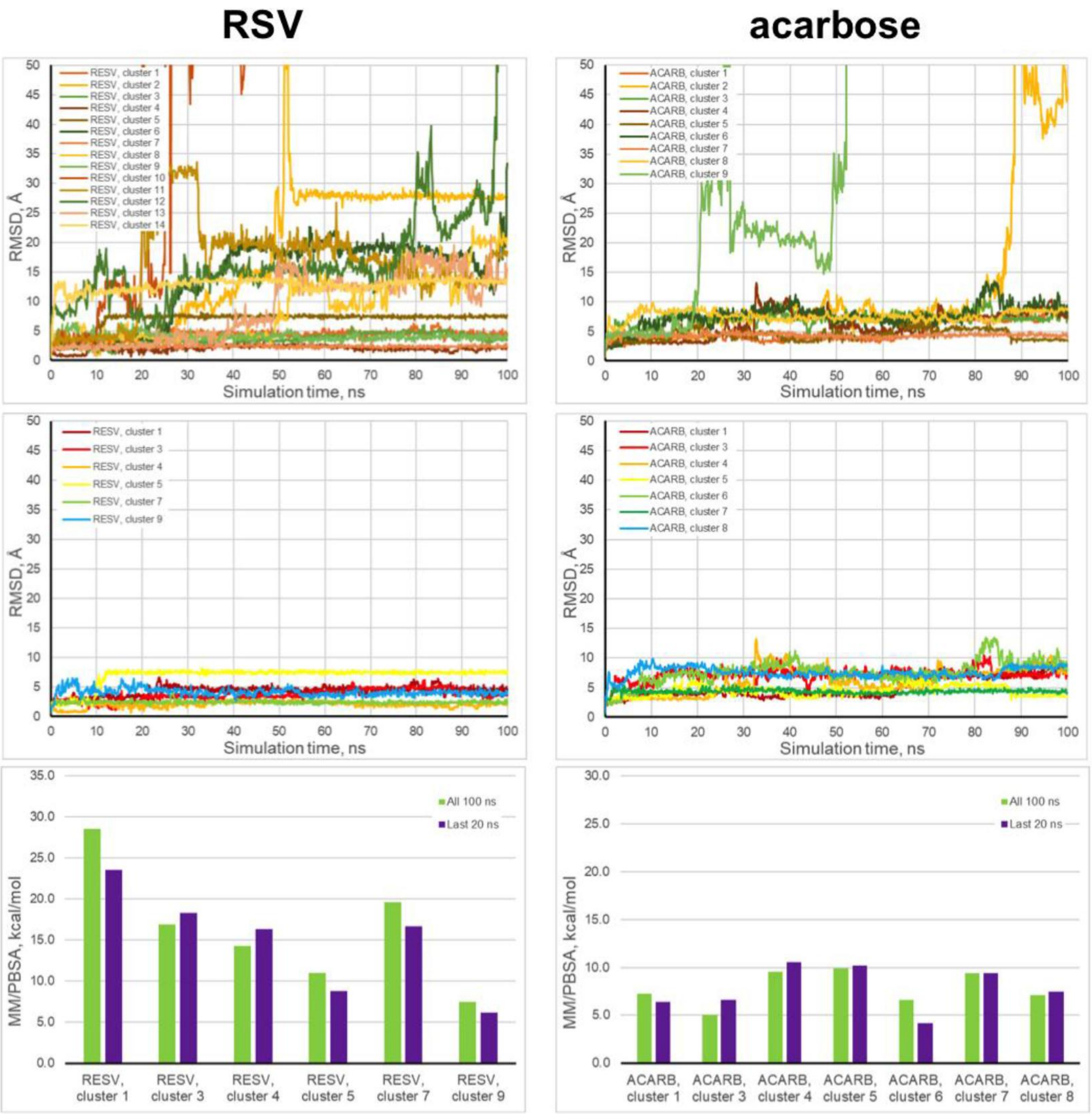

Supplementary Figure 1. Trajectories and MM/PBSA free energy analysis of RSV (left) and acarbose (right) forming complexes with the yeast form of GGA.

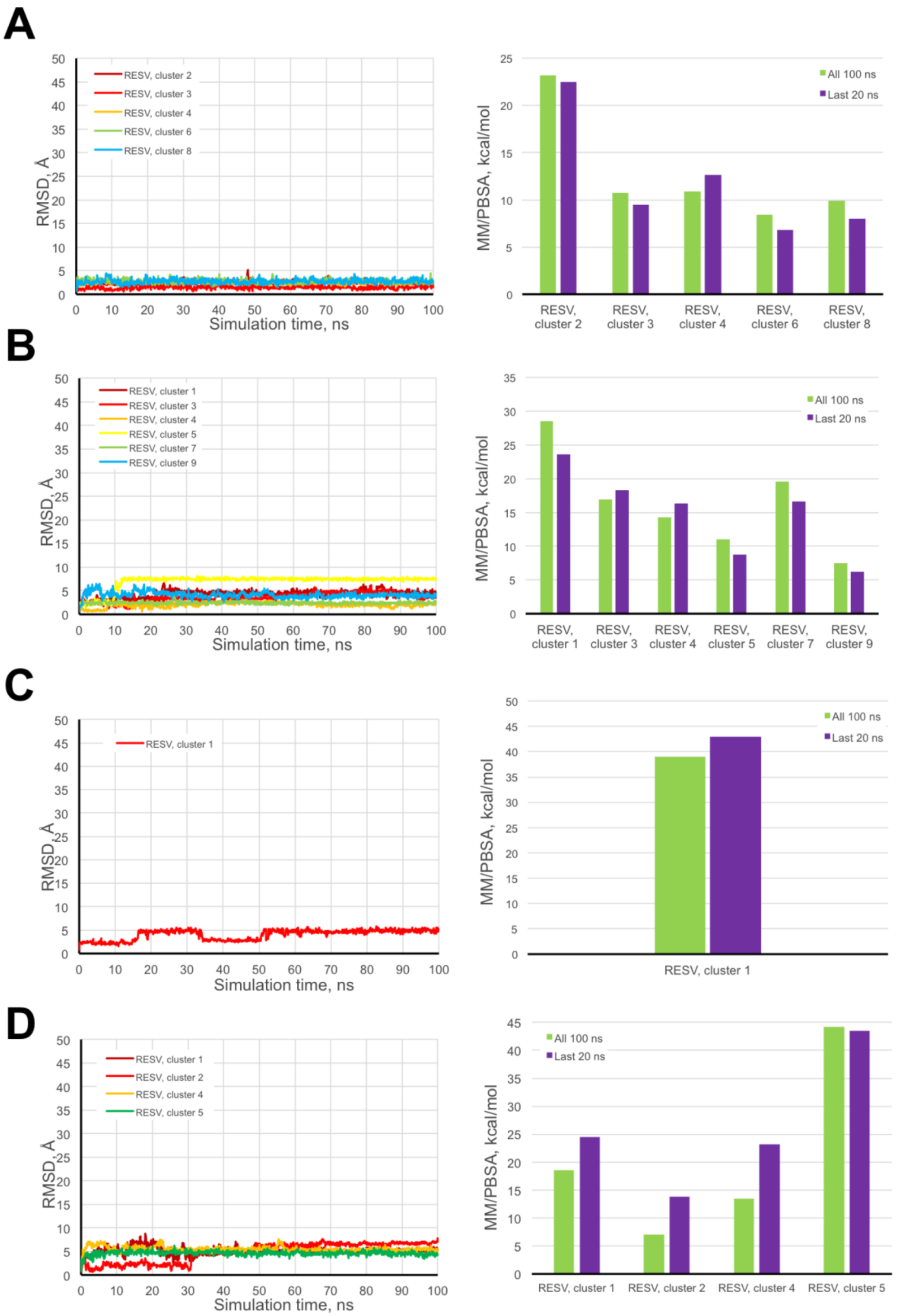

Supplementary Figure 2. Trajectories and MM/PBSA free energy analysis of RSV clusters forming complexes with human GAA (**A**), yeast GGA (**B**), human α-Man I (**C**), and human α-Man II homology model (**D**).
