# Supplementary material for: Resveratrol targets PD-L1 glycosylation and dimerization to enhance antitumor T-cell immunity
Source: Aging (Albany NY). 2020 Jan 4;12(1):8–34. doi: 10.18632/aging.102646 (PMC6977679; doi:10.18632/aging.102646)
Supplement: Supplementary Tables [file aging-12-102646-s001..pdf]

SUPPLEMENTARY TABLES

Supplementary Table S1. Details of the interaction of RSV docked to yeast α-glucosidase.

Cluster number	ΔG, [kcal/mol]	Dissoc. constant, [μM]	Members
1	-7.89	1.650	2.9%
2	-7.43	3.590	4.1%
3	-7.28	4.640	6.9%
4	-7.08	6.420	2.6%
5	-6.78	10.770	1.8%
6	-6.76	11.160	2.3%
7	-6.66	13.160	1.9%
8	-6.64	13.540	1.4%
9	-6.55	15.870	4.1%
10	-6.53	16.310	2.9%
11	-6.43	19.360	3.8%
12	-6.33	22.950	3.8%
13	-6.30	24.190	1.3%
14	-6.27	25.250	3.2%

(Uniprot code: **P53008**, PDB code: **4J5T**). For the best-docked RSV molecule of each cluster, the Gibbs free energy (ΔG, Kcal/mol), the dissociation constant, and the number of molecules members (as %) are shown.

Supplementary Table S2. Details of the interaction of RSV docked to human α-glucosidase.

Cluster number	ΔG, [kcal/mol]	Dissoc. constant, [μM]	Members
1	-8.04	0.128	3.1%
2	-7.78	0.197	1.7%
3	-7.66	0.242	1.4%
4	-7.37	0.399	0.9%
5	-7.34	0.420	1.7%
6	-7.28	0.462	3.0%
7	-7.28	0.461	1.0%
8	-7.02	0.710	2.1%
9	-6.78	1.077	0.8%
10	-6.73	1.166	1.3%
11	-6.72	1.193	1.1%

(Uniprot code: **P10253**, PDB code: **5NN4**). For the best-docked RSV molecule of each cluster, the Gibbs free energy (ΔG, Kcal/mol), the dissociation constant, and the number of molecules members (as %) are shown.

Supplementary Table 3. Details of the interaction of RSV docked to human α-mannosidase I.

Cluster number	ΔG, [kcal/mol]	Dissoc. constant, [μM]	Members
1	-8.25	0.892	2.1%

(Uniprot code: **Q9UKM7**, PDB code: **5KIJ**). For the best-docked RSV molecule of each cluster, the Gibbs free energy (ΔG, Kcal/mol), the dissociation constant, and the number of molecules members (as %) are shown.
